# Self-efficacy and coping style in relation to psychological distress and quality of life in informal caregivers of patients with head and neck cancer: a longitudinal study

**DOI:** 10.1007/s00520-022-07553-x

**Published:** 2023-01-09

**Authors:** Kira S. van Hof, Arta Hoesseini, Irma M. Verdonck-de Leeuw, Femke Jansen, C. René Leemans, Robert P. Takes, Chris H. J. Terhaard, Robert J. Baatenburg de Jong, Aniel Sewnaik, Marinella P. J. Offerman

**Affiliations:** 1 Department of Otorhinolaryngology and Head and Neck Surgery, Erasmus MC Cancer Institute, Erasmus University Medical Center, Dr. Molewaterplein 40, 3015 GD Rotterdam, The Netherlands; 2 Department of Otorhinolaryngology and Head and Neck Surgery, Cancer Center Amsterdam, Amsterdam UMC, Location Vrije Universiteit Amsterdam, De Boelelaan 1117, Amsterdam, The Netherlands; 3 Cancer Center Amsterdam, Treatment and Quality of Life, Amsterdam, The Netherlands; 4 Department of Clinical, Neuro and Developmental Psychology, Vrije Universiteit Amsterdam, Van der Boechorststraat 7–9, Amsterdam, The Netherlands; 5 Amsterdam Public Health, Mental Health, Amsterdam, The Netherlands; 6 Department of Otolaryngology and Head and Neck Surgery, Radboud University Medical Center, Nijmegen, The Netherlands; 7 Department of Radiation Oncology, University Medical Center, Utrecht, The Netherlands

**Keywords:** Informal caregivers, Head and neck cancer, Self-efficacy, Coping, Quality of life, Psychological distress

## Abstract

**Objective:**

In order to understand how informal caregivers of head and neck cancer (HNC) patients deal with the consequences of the disease, we investigated their self-efficacy and coping style in relation to symptoms of anxiety and depression (distress) and quality of life (QoL) over time. In addition, factors associated with self-efficacy and coping style were investigated.

**Methods:**

A total of 222 informal caregivers and their related HNC patients were prospectively followed as part from the multicenter cohort NETherlands QUality of life and Biomedical cohort studies In Cancer (NET-QUBIC). Self-efficacy and coping style were measured at baseline, and distress and QoL at baseline and 3, 6, 12, and 24 months after treatment.

**Results:**

Informal caregivers had a high level of self-efficacy comparable with patients. Caregivers used “seeking social support,” “passive reacting,” and “expression of emotions” more often than patients. Factors associated with self-efficacy and coping were higher age and lower education. Higher self-efficacy was related with better QoL and “active tackling” was associated with less depression symptoms. “Passive reacting” and “expression of emotions” were associated with higher psychological distress and reduced QoL.

**Conclusion:**

Among informal caregivers of HNC patients, higher self-efficacy and “active tackling” were associated with better functioning over time, while “passive reacting” and “expression of negative emotions” were associated with worse functioning. Awareness of the differences in self-efficacy skills and coping and their relationship with QoL and psychological distress will help clinicians to identify caregivers that may benefit from additional support that improve self-efficacy and “active tackling” and reduce negative coping styles.

**Supplementary information:**

The online version contains supplementary material available at 10.1007/s00520-022-07553-x.

## Introduction

Head and neck cancer (HNC) is globally the seventh most frequent cancer type, known for its high morbidity and mortality rates [1–4]. With a 5-year relative survival between 30 and 70%, diagnosis often has a major impact on patients and their loved ones [2, 5, 6]. Both patients and their informal caregivers—most often spouses, but also children or close friends—have to cope with the consequences of the disease and treatment. HNC patients are often most dependent on their informal caregivers for both emotional support and complex practical tasks, which causes a high caregiver burden [7]. This high caregiver burden is associated with higher anxiety and depression levels in caregivers. Informal caregivers may even experience more anxiety symptoms than patients themselves, especially at time of diagnosis and in the first 6 months after treatment [8, 9]. Also, high depression levels of caregivers may negatively influence patients quality of life (QoL) during long-term follow-up [10].To provide optimal support, insight into the way people deal with a certain situation is needed which can be divided in skills of *self-efficacy* and *coping mechanisms. Self-efficacy* has been defined as the confidence in your own ability to influence successful outcomes in challenging or stressful situations [11]. Also in cancer caregivers, the effect of self-efficacy on stress and well-being is studied [12–14]. Chirico et al. found in their meta-analysis that caregivers with high self-efficacy had less psychological distress and a better QoL [15]. However, not much is known about the role of self-efficacy among informal caregivers of HNC patients. One cross-sectional study by Offerman et al. reported on self-efficacy of a specific group of informal caregivers of HNC patients treated with a laryngectomy; higher self-efficacy was associated with reduced psychological distress in caregivers [16].

*Coping* is the way someone responds to challenging or stressful situations [17]. Coping has two major functions: react to change the distressing situation (problem-focused coping) and controlling distressing emotions (emotion-focused coping) [18]. The influence of coping on QoL and psychological distress in general is reported frequently [17]. In caregivers of HNC patients, this relationship was evaluated in a cross-sectional study of Verdonck-de Leeuw et al.; passive coping (i.e., “total withdrawal from social activities”) in HNC caregivers was associated with psychological distress [19]. It is our hypothesis that self-efficacy and coping style are also associated with psychological distress and QoL during long-term follow-up. However, due to the paucity of longitudinal studies, it remains unclear what the role of self-efficacy and coping is in relation to QoL and psychological distress over time, among informal caregivers of HNC patients.^1^

The aims of this prospective longitudinal study were (a) to examine self-efficacy and coping of informal caregivers at time of diagnosis, (b) to identify factors associated with self-efficacy and coping style, and (c) to estimate associations between self-efficacy and coping at baseline and psychological distress and QoL over time.

## Materials and methods

### Setting and participants

The current study used data of a multicenter prospective cohort study the Netherlands Quality of life and Biomedical Cohort study in HNC (NET-QUBIC) [20]. This ongoing cohort study recruited newly diagnosed HNC patients and informal caregivers at five hospitals in the Netherlands between March 2014 and July 2018. Eligible patients were asked if their spouse, family member, or friend wanted to participate as informal caregiver.

### Exclusions and eligibility

Inclusion criteria were patients older than 18 years with a newly diagnosed HNC squamous cell carcinoma that could be treated with curative intend and was not previously treated. In order to understand the questionnaires, both caregivers and patients had to be able to understand, speak, and read the Dutch language. Patients and caregiver couples were excluded if the patients had a malignancy of the salivary glands, nasopharynx, thyroid, skin, or lymphoma in the head and neck region. Another reason for exclusion of patients were severe psychiatric disorders (e.g., schizophrenia, Korsakoff’s syndrome, severe dementia), causing problems with understanding the questionnaires and reliability of the answers. Approval was given by the Medical Ethical Committee of the VU University Medical Center Amsterdam (2013.301(A2018.307)-NL45051.029.13). Further description about the NET-QUBIC cohort is published elsewhere [21].

### Measures

In this study, data at baseline, 3, 6, 12, and 24 months after treatment were used. Both informal caregivers and patients completed patient-reported outcome measures (PROMs).

*Demographic characteristics* were collected with an electronic Case Report form (OpenClinica). Additional information was obtained from the patients’ medical records. To score comorbidity and performance of patients, the ACE-27 comorbidity score and the WHO performance status were used [22, 23].

*Self-efficacy* was measured with the general self-efficacy scale (GSES) [11, 24]. This questionnaire consists of 10 items and investigates whether someone is confident that their actions are leading to successful outcomes in difficult situations. Each item can be scored from 1 (“not at all true”) to 4 (“exactly true”). A higher score represents a higher level of self-efficacy (range 10–40). Reference values of the general Dutch population are a mean score of 31.1 and standard deviation of 5.0 [25]. The scale has a high internal consistency (Cronbach’s alpha coefficients ranging from 0.82 to 0.93) [11].

*Coping* was measured with the Utrecht Coping List (UCL) [26]. This questionnaire consists of 47 items and measures the psychological strategies that people show when facing difficult situations. Seven coping styles are identified, of which one contains a problem-focused coping style: active reacting (i.e., “the intend to solve problems”). The other coping styles are emotion focused: seeking social support (i.e., “discuss problems with family”), palliative reacting (i.e., “looking for distraction”), passive reacting (i.e., “total withdrawal from social activities”), expression of negative emotions (i.e., “the expression of frustration or anger”), avoidance (i.e., “denying the problem”), and reassuring thoughts implies putting things into perspective (i.e., “it could have been worse”). Higher scores suggest a greater appearance of that particular coping mechanism. Psychometric properties were acceptable [26].

*Psychological distress* was measured using the Hospital Anxiety and Depression Scale [27]. This 14-item questionnaire can be divided in the subscales anxiety (HADS-A) and depression (HADS-D), ranging from 0 to 21. Higher scores represent more symptoms of depression or anxiety. Scores of ≥ 8 represent clinical relevant symptoms [28]. The minimal clinical important difference between groups of patients for the HADS is 1.7 [29]. The HADS is a valid instrument (Cronbach’s alpha for the subscales varied from 0.67 to 0.90) [27].

*Quality of life* was measured with the 30-item questionnaire, European Organization for Research and Treatment of Cancer Quality of Life Questionnaire (EORTC-QLQ-C30) [30]. The global quality of life scale was used in the current study. Score can range from 0 to 100, where higher scores represent better QoL. This scale is reliable with a Cronbach’s alpha coefficient of 0.89 [30]. The minimal clinical important difference of QoL subdomain is 8.64 points among HNC patients [31]. Mean QoL scores of 77.4–77.9 are found in Dutch reference data [32, 33].

### Statistical analysis

Analyses were performed using the statistical software program R [34]. Descriptive analyses were performed for baseline characteristics. Mann–Whitney *U* test for continuous data and χ^2^ test for categorical data were used. The paired-samples *T*-test was used to compare GSES and UCL scores at baseline between informal caregivers and patients. To assess the association with baseline characteristics and coping and self-efficacy, multivariate regression analyses with gender, age, education, smoking, and drinking as covariates were performed for each subdomain. Among caregivers, the associations between self-efficacy and coping at baseline with QoL and psychological distress over time were evaluated with mixed effect models from the package nlme [35]. Models were adjusted for gender, age, education, and lifestyle, if needed. Interactions with time were explored, after which the best fitted models were selected with forward selection. We used natural cubic splines to capture the non-linear nature of the outcomes. Graphs were made with ggplot2 to visualize the associations of coping with distress and QoL over time [36]. Mixed models were used as they can handle missing data over time and adjust for correlations in the repeated measures. A two-sided *p*-value of less than 0.05 was considered statistical significant.

## Results

A total of 262 informal caregivers and their related HNC patients were eligible for inclusion. Eventually, 222 couples were included in the current study as 40 informal caregivers did not complete the GSES and UCL (Appendix). Most informal caregivers were spouses (81.5%) and female (73.0%). The same amount of caregivers and patients were smoking at baseline, but a higher amount of patients were former or not daily smokers. More than half of the patients (50.7%) consumed more than six alcohol units per week, compared to 40.1% of the caregivers. All details of the study population can be found in Table 1.

**Table 1:** Descriptive characteristics of informal caregivers and patients

	Patients (*N* = 222) Mean (SD) Frequency (%)	Total no. missing (%)	Caregivers (*N* = 222) Mean (SD) Frequency (%)	Total no. missing (%)	*p*-value
Age, years	63.6 (9.7)	0 (0%)	59.2 (11.4)	0 (0%)	< 0.001
Age, range	35–85	0 (0%)	19–88	0 (0%)	
Gender					< 0.001
Male	168 (75.7%)	0 (0%)	60 (27.0%)	0 (0%)	
Female	54 (24.3%)	0 (0%)	162 (73.0%)	0 (0%)	
Caregiver type				0 (0%)	
Partner			189 (85.1%)		
Daughter/son			25 (11.3%)		
Other			8 (3.6%)		
Education level		13 (5.8%)		3 (1.4%)	0.983
Lower	78 (37.3%)		80 (36.5%)		
Intermediate	59 (28.2%)		62 (28.3%)		
Tertiary	72 (34.4%)		77 (35.2%)		
Smoking		14 (6.3%)		1 (0.5%)	0.005
Yes, daily	37 (17.8%)		37 (16.7%)		
Former/not daily	123 (59.1%)		102 (46.2%)		
Never	48 (23.1%)		82 (37.1%)		
Alcohol/week		13 (5.9%)		5 (2.3%)	0.088
0 per week	58 (27.8%)		73 (33.6%)		
1–6 per week	45 (21.5%)		57 (26.3%)		
> 6 per week	106 (50.7%)		87 (40.1%)		
Tumor site		0 (0%)			
Oral cavity	65 (29.3%)				
Oropharynx	73 (32.9%)				
Hypopharynx	13 (5.9%)				
Larynx	63 (28.4%)				
Unknown primary	8 (3.6%)				
Disease stage		0 (0%)			
I	54 (24.3%)				
II	42 (18.9%)				
III	36 (16.2%)				
IV	90 (40.5%)				
WHO performance		0 (0%)			
0	167 (75.2%)				
I–II	55 (24.8%)				
Comorbidity (ACE-27-baseline)		16 (7.2%)			
None	60 (29.1%)				
Mild	81 (39.3%)				
Moderate	41 (19.9%)				
Severe	24 (11.7%)				

### Self-efficacy and coping style

In Table 2, results on self-efficacy and coping style of informal caregivers and patients at baseline are shown. Informal caregivers had a level of self-efficacy (mean 31.8, SD: 4.6) comparable to patients (mean 32.5, SD 5.4, *p* = 0.145) and the general Dutch population (mean 31.1, SD 5.0). Regarding coping style, informal caregivers scored higher on the emotion-focused coping styles compared to patients: “seeking social support” (*p* = 0.034), “passive reacting” (*p* = 0.016), and “expression of negative emotions” (*p* < 0.001).

**Table 2:** Coping mechanisms and self-efficacy in informal caregivers and patients

	Explanation	Range	Caregivers (*N* = 222) Mean (SD)	Patients (*N* = 210) Mean (SD)	*p*-value
Self-efficacy	“The belief that your own behavior leads to the desired outcome”	10–40	31.8 (4.6)	32.5 (5.4)	0.145
Coping					
Active tackling	“Trying to solve the problem”	7–28	18.9 (3.3)	18.8 (3.8)	0.691
Seek social support	“Sharing concerns or getting help”	6–24	13.7 (3.3)	13.1 (3.3)	0.034
Palliative reacting	“Looking for distraction”	8–32	17.3 (3.1)	17.2 (3.4)	0.631
Passive reacting	“Withdrawal from social activities”	7–28	10.7 (2.8)	10.0 (2.6)	0.016
Expression of emotions	“Showing frustration or anger”	3–12	5.6 (1.4)	4.8 (1.4)	0.001
Avoiding	“Keep away from the difficult situation”	7–28	15.4 (2.7)	15.2 (3.2)	0.568
Reassuring thoughts	“Seeing things in a positive light”	5–20	12.6 (2.1)	12.3 (2.4)	0.143

Self-efficacy was measured with the General Self-Efficacy Scale (GSES) and coping style with the Utrecht Coping List (UCL)

### Variables associated with self-efficacy and coping style

Lower education level was associated with less self-efficacy, “active reacting,” “palliative reacting,” and “seeking social support” (Table 3). Higher age was associated with less “active tackling” and “seeking social support.” Female gender was associated with more “palliative reacting” and “seeking social support.” Lifestyle variables as smoking and drinking were not related with coping style or self-efficacy in our cohort.

**Table 3:** Variables associated with self-efficacy and coping styles

Caregivers	Baseline variable		β	SE	*p*-value	OR
Self-efficacy	Education	Low	“			
		Intermediate	2.02	0.82	0.014	7.55
		High	0.89	0.82	0.283	2.83
Coping						
Active tackling	Age		− 0.04	0.02	0.035	–
	Education	Low	“			
		Intermediate	2.51	0.52	< 0.001	12.29
		High	2.88	0.52	< 0.001	17.77
Seek social support	Age		− 0.07	0.02	< 0.001	–
	Gender	Male	“			
		Female	2.00	0.47	< 0.001	7.36
	Education	Low	“			
		Intermediate	1.27	0.51	0.013	3.57
		High	2.80	0.51	< 0.001	16.49
Palliative reacting	Gender	Male	“			
		Female	1.55	0.49	0.002	4.72
	Education	Low	“			
		Intermediate	0.97	0.53	0.071	2.64
		High	1.30	0.54	0.016	3.67
Passive reacting	–					
Expression of emotions	–					
Avoiding	–					
Reassuring thoughts	–					

Variables associated with coping style and self-efficacy in informal caregivers. Multivariate regression analysis with gender, age, education, smoking, drinking, and caregiver cancer in past as covariates

### Associations between baseline self-efficacy and coping style and distress and QoL over time

**Fig. 1:**
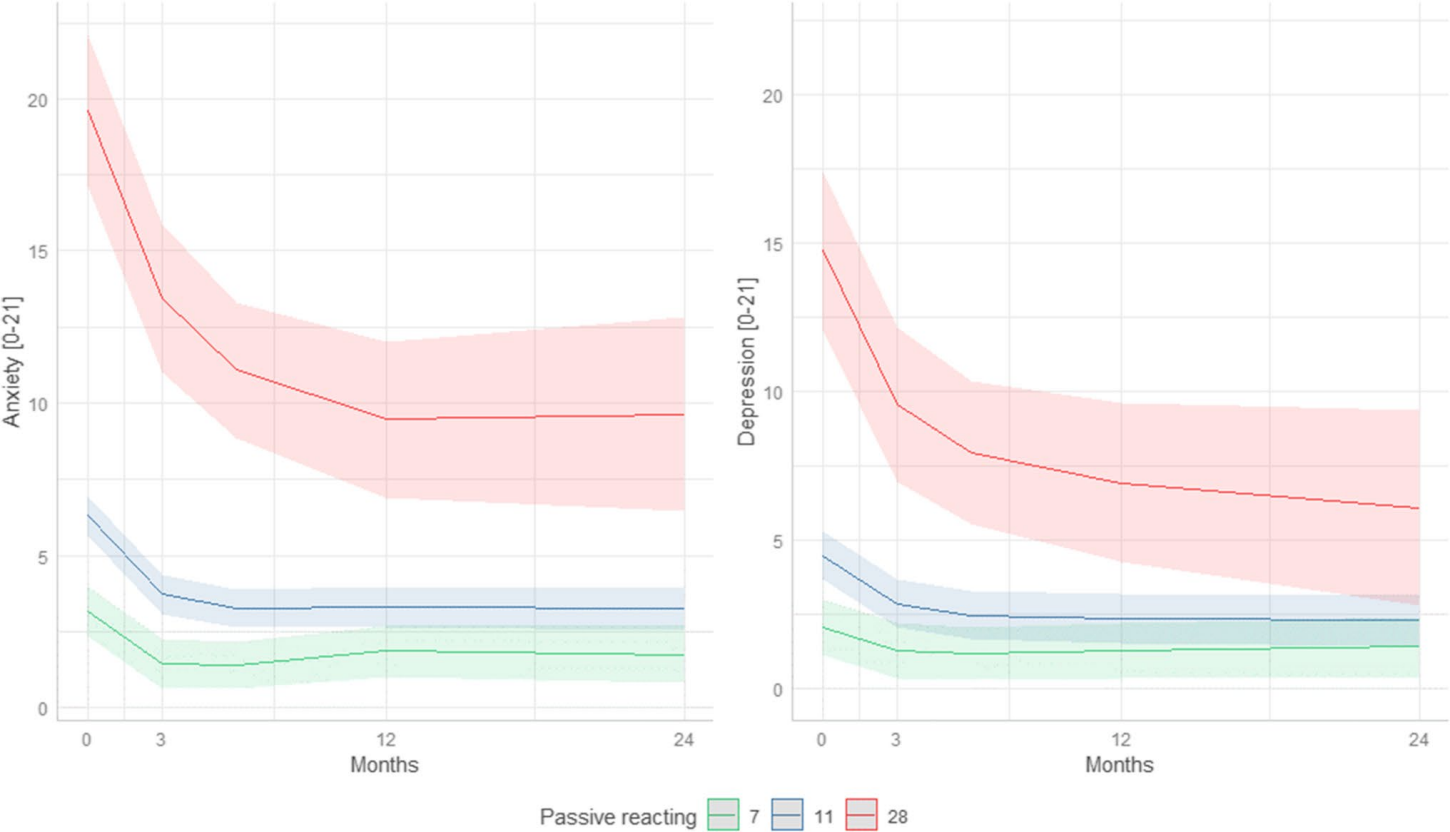
Graphical representation of the associations between the coping style “passive reacting” at baseline and the course of symptoms of anxiety and depression over time. The red line indicates the anxiety and depression symptoms over time when at baseline passive reacting was the highest score (UCL passive reacting = 28), the blue line indicates the mean passive reacting score (UCL passive reacting = 11), and the green line indicates no use of passive reaction (UCL passive reacting = 7). The 95% confidence intervals of the predicted anxiety and depression scores are presented in the lighter color

**Fig. 2:**
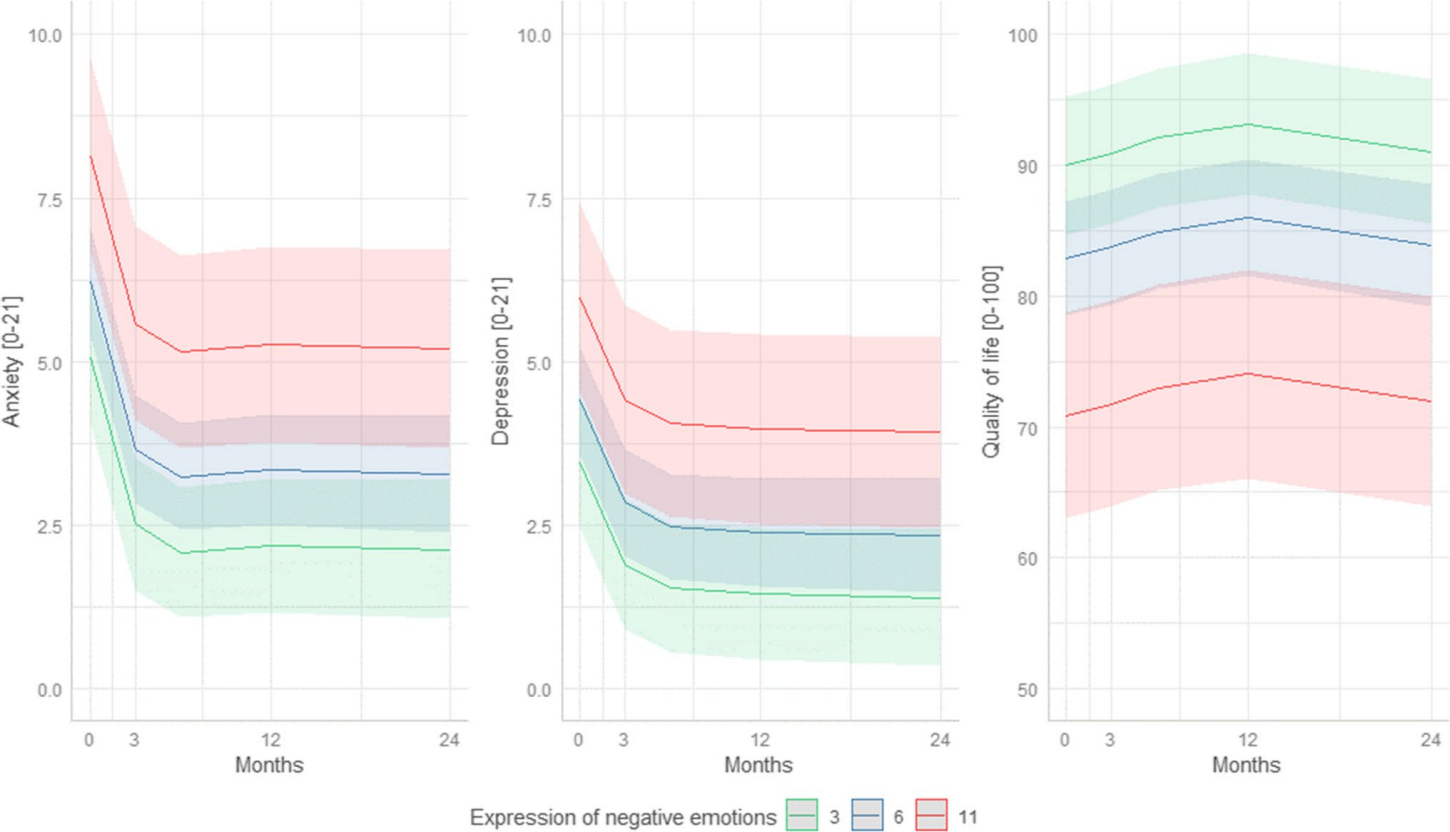
Graphical representation of the associations between the coping style expressing negative emotions and symptoms of anxiety and depression, and QoL over time. The red line indicates the anxiety and depression symptoms and QoL over time when at baseline expression of negative emotions was the highest score (UCL expression of emotions = 11), the blue line indicates the mean (UCL expression of emotions = 6), and the green line indicates no use of expression of negative emotions (UCL expression of emotions = 3). The 95% confidence intervals of the predicted anxiety and depression and QoL scores are presented in the lighter color

As shown in Table 4, a higher level of self-efficacy was significantly associated with better QoL over time (*p* < 0.012). A higher level of the coping style “active tackling” was associated with less symptoms of depression over time (*p* < 0.001). A higher level of the coping style “expression of negative emotions” was associated with more symptoms of anxiety and depression and a worse QoL over time (*p* < 0.005). Furthermore, a higher level of the coping style “passive reacting” was related with more symptoms of anxiety and depression (*p* < 0.001). Figures 1 and 2 depict the results graphically. Symptoms of anxiety and depression were higher from baseline to two years after treatment in patients using the coping style “passive reacting” (Fig. 1). Depression symptoms of caregivers with high “passive reacting” declined during long-term follow-up, while caregivers without “passive reacting” had more stable and lower depression symptoms. Two years after treatment, differences in depression symptoms were still present. In Fig. 2, the significant association between “expression of negative emotions” at baseline and QoL and psychological distress is visualized. When caregivers used the coping style “expression of negative emotions” at baseline, this was associated with higher psychological distress and lower QoL. When caregivers with high “expression of negative emotions” were compared to caregivers without expression of emotions, a large difference was observed for QoL, anxiety, and depression at all measurement moments (Fig. 2)*.*

**Table 4:** The course of symptoms of anxiety and depression and quality of life in relation to self-efficacy coping style in caregivers

Outcome	Baseline variable	β	SE	*p*-value
Anxiety	Passive reacting	0.78	0.07	< 0.001
	Expression of negative emotions	0.38	0.12	< 0.001
Depression	Active tackling	− 0.15	0.05	0.004
	Passive reacting	0.60	0.07	< 0.001
	Expression of negative emotions	0.32	0.11	0.005
Global QoL	Self-efficacy	0.48	0.19	0.012
	Expression of negative emotions	− 2.39	0.61	0.001

The association between self-efficacy and coping styles at baseline and anxiety, depression, and quality of life over time. Linear mixed model analyses were performed with UCL and GSES outcomes at baseline as covariates. Models were adjusted for the confounders gender, age, and education. Through forward selection, only significant variables were put in the final models

## Discussion

In this prospective longitudinal cohort study, we investigated the level of self-efficacy and coping style among informal caregivers, which are mainly spouses. The average level of self-efficacy was comparable in informal caregivers and HNC patients. High levels of self-efficacy at baseline were associated with better quality of life (QoL) at all measurement moments. Furthermore, we found that informal caregivers used the negative emotion-focused coping mechanisms “passive reacting” and “expression of negative emotions” more often than patients. Used coping mechanisms at baseline were associated with psychological distress and QoL at all measurement moments. More specifically, “active tackling” was associated with less depressive symptoms and “passive reacting” and “expression of negative emotions” with more psychological distress and reduced QoL at all measurement moments.

Both informal caregivers and HNC patients had a high level of self-efficacy, which was comparable to the general Dutch population (mean GSES scores 31.8, 32.5, and 31.1, respectively) [25]. Another study in a cohort of informal caregivers of cancer patients in general also found that self-efficacy of informal caregivers and patients was comparable. The levels of self-efficacy in that study (mean GSES scores 30.2 and 29.2, respectively) were also similar to the study population in the current study [37]. Self-efficacy was significantly associated with educational level but not with age, gender, or lifestyle. Regarding coping style, the informal caregivers in this study used the negative emotion-focused coping styles “passive reacting” and “expression of negative emotions” more often than patients. Informal caregivers in our cohort used “passive reacting” as often as informal caregivers of children with cancer. Compared to a total different group of caregivers of patients with a bipolar disorder, our caregivers used “passive reacting” less often [38, 39]. This may be explained by the fact that informal caregivers of patients with a bipolar disorder have to cope with a lifetime of deviant behavior of the patient. Passive coping may therefore be used as protection for disappointment. Furthermore, providing care for a family member with psychiatric disorders gives an increased risk for mental health conditions of the caregiver [40]. Caregivers used “expression of negative emotions” approximately as often as caregivers of patients with other diseases and of children with cancer [38, 39]. Coping style was significantly associated with age (“active tackling” and “seeking social support”), gender (“palliative reacting” and “seeking social support”), and educational level (“active reacting,” “palliative reacting,” and “seeking social support”) but not with lifestyle. The association between age and coping style was also found in the study of Derks et al. in which they compared the effect of coping style in younger and older HNC patients [41]. In literature, the relation between female gender and “seeking social support” in HNC patients has been described as well [17]. The finding that lower education level was associated with less self-efficacy and “active tackling” confirmed a study by Hütter et al. who found that lower education was related to more “depressive coping” in a cohort of patients with closed head injuries [42]. A significant association between lower education and passive coping was found in the general population as well [43]. Brouwer et al. suggested that people with a lower educational level use a passive coping style to meet immediate needs, not overseeing the negative long-term consequences [43]. None of the baseline characteristics were significantly associated with “passive reacting” and “expression of negative emotions” (the coping styles that caregivers used more often compared to patients).

In the current study, we estimated the role of self-efficacy and coping style as assessed at baseline on the course of distress and QoL over time among informal caregivers of HNC patients. Corrected for educational level, a higher level of self-efficacy at baseline was significantly associated with better QoL at all measurement moments from baseline to two years after treatment. In a cross-sectional study of Offerman et al. among caregivers of HNC patients after laryngectomy, a significant association between higher self-efficacy and lower psychological distress was found as well [16]. Hampton et al. argued that self-efficacy has the potential to be a stress mediator in a cohort of informal caregivers of cancer patients during end-of-life care [44]. Corrected for the abovementioned significant associations between coping styles and age, gender, and educational level, coping style was significantly associated with distress and QoL at all measurement moments. More specifically, “active tackling” was associated with less depressive symptoms, and “passive reacting” and “expression of negative emotions” were associated with more distress and lower QoL at all measurement moments. In a cross-sectional study by Verdonck-de Leeuw et al., a significant relation between “passive reacting” and psychological distress was found as well [19]. Also, the review of Morris et al. reported on a comparable concept “disengagement coping”: “*an orientation towards drawing attention away from stress, and making an effort to distance oneself from the stressor or related feelings*,” which was related with higher psychological distress in HNC patients [45]. In other studies in HNC patients, the coping style “avoidance” was also found to be associated with more psychological distress and lower QoL, but this was not confirmed in the present study [17, 41, 46]. This can be explained by the correlation that was found in our cohort between “avoiding” and “passive reacting.” After forward selection of our mixed models, “avoiding” was not significantly associated with distress or QoL anymore.

### Strengths and limitations

A major strength of this study is the large prospective cohort of both informal caregivers and their related HNC patients, which was followed throughout the cancer trajectory with repeated measurements from baseline to two years after treatment. This gave us the opportunity to assess the relation between self-efficacy and coping style at baseline with the course of psychological distress and QoL over time, while up to now only few cross-sectional studies reported on this relation [16, 19]. A limitation is that during follow-up, a part of the participants were lost to follow-up (66% of the couples completed the measurements at 24 months after treatment). The only significant difference between the patients who completed the last measurement moment and the drop-out patients was WHO performance; patients who dropped out had a significant worse WHO performance (*p* = 0.006). All other baseline variables were not significantly different. To handle missing data and use all available data, linear mixed-effect models for repeated measurements were used. As no appropriate reference group for the UCL in the general population is available, we were only able to compare the coping style of informal caregivers with their related patients. Furthermore, in most studies, other questionnaires were used to evaluate self-efficacy and coping mechanisms, which makes it difficult to compare our findings to other informal caregiver groups [45]. Another limitation is that dyadic coping was not measured with a specific questionnaire as the Dyadic Coping Inventory, which is still an underexposed topic in literature [47, 48]. Last, we cannot state any conclusions about all caregivers of HNC patients, as patients with sinonasal and parotic malignancies were excluded.

### Clinical implications and future perspectives

Informal caregivers of HNC patients with lower self-efficacy skills and those who mainly use the coping styles “passive reacting” or “expression of negative emotions” at baseline are vulnerable for lower QoL and higher psychological distress from baseline up to long-term follow-up. Therefore, it is important to structurally screen this group of informal caregivers and refer them for (psychological) support when needed. We think that psychological interventions that improve self-efficacy and “active tackling” and reduce “passive reacting” and “expression of negative emotions” will help informal caregivers to function better over time. When addressing self-efficacy and coping style, special attention should be given to caregivers with higher age, females, and/or lower education, as these factors were associated with less self-efficacy and “active tackling.” Providing psychological interventions is not only important for the caregivers themselves, but also for the HNC patients, as in a prior study we found that reduced functioning of informal caregivers was associated with reduced functioning of patients related to them [10]. In a review of Cheng et al., short-term effects of psychoeducational interventions were found on caregivers’ psychological distress, quality of life, caregiver burden, and self-efficacy [49]. Future studies should focus on the long-term impact of these interventions.

## Conclusions

This study contributes to better understand the role of self-efficacy and coping style among informal caregivers and the HNC patients related to them. Higher levels of self-efficacy and “active tackling” at baseline were associated with better global QoL and less symptoms of depression at all measurement moments. The negative coping mechanisms “passive reacting” and “expression of negative emotions” at baseline were associated with more anxiety and depression symptoms from baseline up to two years after treatment. Awareness of the differences in self-efficacy and coping and their relationship with distress and QoL will help clinicians to identify caregivers at risk that may benefit from additional support. We think that (psychological) interventions that improve self-efficacy and “active tackling” and reduce “passive reacting” and “expression of negative emotions” may be helpful to reduce psychological distress and improve QoL, even at long-term follow-up.

## Supplementary information

Below is the link to the electronic supplementary material.

Supplementary file1 (DOCX 36 KB)

## Data Availability

Data is not available as it is part of the ongoing multicenter cohort study NET-QUBIC.

## Footnotes

1 Only squamous cell carcinomas in the oral cavity, oropharynx, hypopharynx, larynx, and unknown primary were included. Carcinomas of the nasopharynx and parotid gland were not included in this cohort.
