# Supplementary material for: Self-efficacy and coping style in relation to psychological distress and quality of life in informal caregivers of patients with head and neck cancer: a longitudinal study
Source: Support Care Cancer. 2023 Jan 9;31(2):104. doi: 10.1007/s00520-022-07553-x (PMC9829635; doi:10.1007/s00520-022-07553-x)
Supplement: Supplementary file 1 — Supplementary file1 (DOCX 36 KB) [file 520_2022_7553_MOESM1_ESM.docx]

**Appendix A: Flowchart of all eligible HNC patients and caregivers and reasons for non-participation**

NET-QUBIC patients

N = 739

NET-QUBIC dyads

(informal caregiver + patient)

N = 262

No participating informal caregivers (n = 477)

NET-QUBIC dyads

N = 253

BASELINE ASSESSMENT

PROMs available

N = 186

3 MONTHS FOLLOW-UP

PROMs available

N = 163

6 MONTHS FOLLOW-UP

PROMs available

N = 153

12 MONTHS FOLLOW-UP

PROMs available

N = 128

24 MONTHS FOLLOW-UP

Dropped-out (n = 9):

- Drop-out patient: physical reasons (n = 1)
- Drop-out patient: psychological reasons (n =2)
- Drop-out patient: logistic reasons (n = 1)
- Drop-out caregiver: no time (n = 1)
- Drop-out caregiver: unknown (n = 4)

Dropped-out (n = 26):

- Patient died (n = 4)
- Caregiver died (n = 1)
- Drop-out patient: physical reasons (n = 2)
- Drop-out patient: psychological reasons (n =2)
- Drop-out patient: logistic reasons (n = 1)
- Drop-out patient: does not feel like it (n = 2)
- Drop-out patient: unknown (n = 3)
- Drop-out caregiver: psychological reasons (n = 1)
- Drop-out caregiver: logistic reasons (n = 1)
- Drop-out caregiver: does not feel like it (n = 1)
- Drop-out caregiver: relationship changed (n = 1)
- Drop-out caregiver: unknown (n = 7)

Missing data M3 (n = 10)

Dropped-out (n = 20):

- Patient died (n = 5)
- Drop-out patient: does not feel like it (n = 1)
- Drop-out caregiver: physical reasons (n = 1)
- Drop-out caregiver: no time (n = 1)
- Drop-out caregiver: logistic reasons (n = 4)
- Drop-out caregiver: does not feel like it (n = 4)
- Drop-out caregiver: unknown (n = 4)

Missing data M6 (n = 13)

Dropped-out (n = 16):

- Patient died (n = 6)
- Drop-out patient: physical reasons (n = 2)
- Drop-out patient: psychological reasons (n = 1)
- Drop-out caregiver: does not feel like it (n = 1)
- Drop-out caregiver: unknown (n = 6)

Missing data M12 (n = 7)

Dropped-out (n = 23):

- Patient died (n = 6)
- Caregiver died (n = 1)
- Drop-out patient: physical reasons (n = 3)
- Drop-out patient: psychological reasons (n = 2)
- Drop-out caregiver: physical reasons (n = 2)
- Drop-out caregiver: does not feel like it (n = 1)
- Drop-out caregiver: unknown (n = 8)

Missing data M24 (n = 9)

Total response rate = 84%

Response rate dyads both alive = 86%

PROMs available

N = 222

Total response rate = 73%

Response rate dyads both alive = 77%

Total response rate = 69%

Response rate dyads both alive = 74%

Total response rate = 58%

Response rate dyads both alive = 66%
